# Comparison of outcomes of pedicled jejunal and colonic conduit for esophageal reconstruction

**DOI:** 10.1186/s12893-020-00810-y

**Published:** 2020-07-16

**Authors:** Sicong Jiang, Changying Guo, Bin Zou, Jianguo Xie, Zhihui Xiong, Yukang Kuang, Jianjun Tang

**Affiliations:** 1 Department of Thoracic Surgery, Jiangxi Cancer Hospital of Nanchang University, No. 519 Beijing East Road, Nanchang, 330006 Jiangxi China; 2 Department of Obstetrics, Tongde Hospital of Zhejiang Provience, Zhejiang, 310012 Hangzhou China; 3 Department of Respiratory and Critical Care Medicine, The First Affiliated Hospital of Nanchang University, Nanchang, 330006 Jiangxi China

**Keywords:** Esophageal cancer, Reconstruction, Gastric remnant, Pedicle jejunum, Postoperative

## Abstract

**Background:**

At present, the gastric tube is the first choice for esophageal reconstruction after esophagectomy for various benign and malignant diseases. However, when the stomach is not available, a pedicled jejunum or colon is used to reconstruct the esophagus. The present study aimed to compare the postoperative outcomes and quality of life of patients receiving jejunal and colonic conduits.

**Methods:**

In the present retrospective study, the clinical data of 71 patients with esophageal carcinoma, who received jejunal reconstruction (jejunum group, *n* = 34) and colonic reconstruction (colon group, *n* = 37) from 2005 to 2015, were compared.

**Results:**

Compared with the colon group, the jejunum group had a lower incidence of postoperative anastomotic leakage, lesser duration of postoperative drainage, and faster recovery. Furthermore, the scores were better in the jejunum group than in the colon group, in terms of short-term overall quality of life, physical function and social relationships. Moreover, the jejunal group had a significantly lower frequency of pH < 4 simultaneous reflux time > 5 min (N45) and the longest reflux time (LT) at 24 weeks after surgery.

**Conclusion:**

In esophageal cancer, when gastric tube construction is not feasible, a pedicled jejunum may be preferred over a colonic conduit due to lower incidence of acid reflux, anastomotic leakage and higher postoperative short-term quality of life, and rapid postoperative recovery.

## Background

The incidence of esophageal carcinoma is gradually increasing, and has become the fifth most common cancer in China [1]. Surgical resection remains the main component of multimodality treatment for esophageal cancer. After esophageal resection, the gastric tube is the most preferred primary conduit for reconstruction [2]. Recently, the number of patients with esophageal cancer after gastrectomy has gradually increased. This phenomenon may be due to the increasing survival of patients after gastric resection, the changes in dietary habits after gastrectomy, or the development of gastroesophageal reflux disease [3, 4]. In cases with prior gastrectomy, the stomach may not be available for reconstruction, and a jejunal or colonic conduit may have to be used [5].

Esophagectomy with pedicled colonic reconstruction was first reported by Kelling from Germany in 1911 [6], in which a pedicled transverse colon was chosen to reconstruct the lower esophagus. Since the colonic mesentery is very long, the colon has good ductility and mobility, which significantly reduces the anastomotic tension [7, 8]. In 1946, Longmire and Ravitch [9] of Johns Hopkins Hospital in the United States first reported the use of a pedicled jejunum as an esophageal substitute. However, since the mobility of a pedicled jejunum is limited, it has not been widely used in clinical practice for esophageal reconstruction. However, with the improvement of surgical techniques and perioperative management, many institutional centers have begun to use a pedicled jejunum for esophageal reconstruction in recent years [10]. The reasons behind the use of a jejunum are as follows: its luminal diameter is similar to that of esophagus; it has rich blood supply; it exerts minimal pressure on the surrounding chest organs; it has peristalsis, which is conducive for food propulsion; it causes lesser halitosis, when compared to patients receiving a colonic conduit; there is no requirement for strict bowel preparation before surgery when jejunal conduit is planned for reconstruction [11, 12].

Very few studies have reported the outcomes of jejunal and colonic esophageal reconstruction, with most of these studies having a small sample size [13–15]. Therefore, the choice of a jejunum or colon for esophageal reconstruction remains controversial, and needs further research. The main purpose of the present study was to compare the intraoperative and short-term outcomes of colonic and pedicled jejunal conduits for esophageal reconstruction by comparing postoperative severe complications and quality of life (QOL).

## Methods

### Patients

The present study retrospectively analyzed a prospectively maintained database of adult esophageal cancer patients with the previous history of gastrectomy, who underwent radical esophageal resection with reconstruction using a pedicled jejunal or colonic conduit at the Department of Thoracic Surgery, Jiangxi Province Tumor Hospital, Nanchang, China from April 2005 to June 2015. Patients with locally advanced esophageal cancer, and patients who had jejunal or colonic lesions, including tumors and inflammatory lesions, were excluded. In addition, patients in poor general condition, who could not tolerate the surgery, and those with severe cardiopulmonary dysfunction and uncontrolled diabetes were excluded. Furthermore, patients who received neoadjuvant therapy were excluded to eliminate the effects of chemoradiation on the outcomes. The present study was approved by the local institutional ethics committee of Jiangxi Province Tumor Hospital. All patients provided a written informed consent prior to the procedure.

### Surgical procedures

**Fig. 1:**
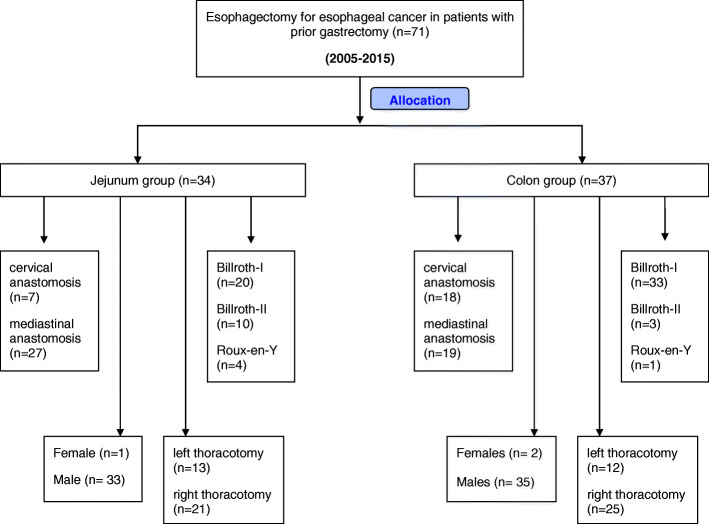
Flow chart showing the various surgical approaches, conduits and types of gastrointestinal reconstruction performed for the study patients

In the present study, 25 patients initially underwent radical esophagectomy through left thoracotomy. Among these patients, 13 patients received jejunal replacement, while 12 patients underwent colonic replacement. The remaining 46 patients underwent surgery through right thoracotomy, with jejunum replacement in 21 cases and colonic replacement in 25 cases (Fig. 1). A total of 25 patients underwent cervical anastomosis, including seven patients in the jejunum group and 18 patients in the colon group (Supplementary Figure 1). All patients underwent mediastinal lymph node dissection and upper abdominal lymph node dissection (including the perigastric and celiac nodes). Cervical node dissection was performed for patients with upper esophageal tumors, and patients with upper mediastinal node metastasis [16].

First, the mediastinal esophagus was completely mobilized through the chest incision in the lateral position, and the incision was closed. Subsequently, the patient was placed in the supine position, an upper abdominal incision was performed, and the mesenteric and vascular arch of the residual stomach and intestines was examined. The choice of conduit was decided by the operating surgeon based on surgical experience, the anatomy of the jejunum and colon, and the location of the anastomosis. The detailed steps of the reconstruction are provided in the appendix.

### Postoperative short-term quality of life evaluation

The present study used a standard questionnaire for the QOL assessment of cancer patients prepared by the European Organization for Research on Treatment of Cancer (EORTC QLQ-C30) [17, 18], and a supplementary scale that specifically assessed the QOL of patients with esophageal cancer (QLQ-OES18) [19]. Based on EORTC multi-center clinical trials and tests, the combination of QLQ-C30 and QLQ-OESl8 was found to more accurately reflect the degree of association between the symptoms of esophageal cancer patients and their QOL [20]. The EORTC questionnaire QLQ-C30 (v.3.0) consisted of 30 items (two for overall QOL, five for physical functions, four for emotional functions, two for social relationships, two for cognitive functions, two for role functions, three for fatigue symptoms, two for nausea and vomiting, two for pain, and one for dyspnea, insomnia, loss of appetite, constipation, diarrhea and economic hardship). The 24-h pH determination of the remnant esophagus in these two groups was performed at 1, 4, 12 and 24 weeks after surgery. These two main observation indicators include the number of times pH < 4 was recorded for > 5 min (N45) and the longest reflux time (LT).

### Statistical analysis

All data analyses were performed using SPSS 23.0 statistical software. Continuous data with normal distribution were expressed as mean ± standard deviation, while those with non-normal distribution were expressed in median (interquartile range). Categorical data were expressed in percentage (%). The QLQ-C30 and QLQ-OES18 scores at different time points, and N45 and LT at different time points were analyzed by repeated measures analysis of variance (ANOVA). *X*^*2*^**-**test was used for categorical data. The grading data in the patient characteristics was compared by rank sum test. Measurement data that conformed to the normal distribution were compared by independent sample *t*-test. A *P*-value of < 0.05 was considered statistically significant.

## Results

### Patient demographics

A total of 71 patients with esophageal cancer underwent radical esophageal resection and reconstruction with the jejunum/colon at our department from 2005 and 2015. None of these patients received preoperative neoadjuvant chemotherapy, while 46 patients received standardized adjuvant chemotherapy after surgery. Patients in the jejunum group were older than patients in the colon group (*P* = 0.02). Ten patients (14%) had hypertension and coronary heart disease. One patient (1.4%) had a history of schizophrenia. Sixty-six patients (92.9%) underwent partial gastrectomy (five patients had gastric cardia cancer, 13 patients had peptic ulcer, and 48 patients had gastric cancer). The remaining five patients underwent total gastrectomy for gastric cancer. The reconstruction techniques for the gastrectomy included Billroth I in 53 cases (jejunum group, 20; colon group, 33), Billroth II in 13 cases (jejunum group, 10; colon group, 3), and Roux-En-Y in five cases (jejunum group, 4; colon group, 1) (Fig. 1). There was no significant difference in tumor pathological type, thoracotomy and N stage between these two groups (Table 1).

**Table 1:** Patient characteristics

	Jejunum group (*n* = 34)	Colon group (*n* = 37)	*P*-value
**Gender**			> 0.99^a^
Male	33 (97.1%)	35 (94.6%)	
Female	1 (2.9%)	2 (5.4%)	
**Age (mean ± SD)**	65.2 ± 7.8	61 ± 6.8	0.02^b^
**Pathological type**			0.31^a^
Squamous cell carcinoma	22 (64.7%)	28 (75.7%)	
Adenocarcinoma	12 (35.3%)	9 (24.3%)	
**Location**			0.04^a^
Upper	6 (17.6%)	11 (29.7%)	
Middle	5 (14.7%)	12 (32.4%)	
Low	23 (67.6%)	14 (37.8%)	
**Anastomotic position**			0.01^a^
Neck	7 (20.6%)	18 (48.6%)	
**Side of thoracotomy**			0.61^a^
Left	13 (38.2%)	12 (32.4%)	
Right	21 (61.8%)	25 (67.6%)	
**Previous operation procedure**			0.01^e^
Billroth-I	20 (58.8%)	33 (89.2%)	
Billroth-II	10 (29.4%)	3 (8.1%)	
Roux-en-Y	4 (11.8%)	1 (2.7%)	
**Pathological T-stage**			0.03^d^
T1	2 (5.9%)	5 (13.5%)	
T2	8 (23.5%)	17 (45.9%)	
T3	20 (58.8%)	12 (32.4%)	
T4	4 (11.8%)	3 (8.1%)	
**Pathological N-stage**			0.52^d^
N0	18 (52.9%)	23 (62.2%)	
N1	7 (20.6%)	10 (27%)	
N2	5 (14.7%)	4 (10.8%)	
N3	4 (11.8%)	0	
**Cancer stage**			0.42^d^
I	3 (8.8%)	6 (15.4%)	
II	14 (41.2%)	17 (43.6%)	
III	15 (44.1%)	14 (35.9%)	
IV	2 (5.9%)	2 (5.1%)	

^a^ χ^2^ test

^b^ Independent Sample T-test

^e^ Monte Carlo method

^d^ Rank sum test

### Operative data

There was a statistically significant difference in terms of the location of the anastomosis between these two groups. However, there was no statistical difference between the two groups in terms of the time interval between the previous gastrectomy and esophagectomy. The number of patients in the jejunum group who received neck anastomosis was significantly fewer than that in the colon group (20.6% vs. 48.6%, *P* = 0.012). The operative time in the jejunum group was shorter than that in the colon group, but the difference was not statistically significant (397.2 ± 107.1 min vs. 415.8 ± 123.8 min, *P* = 0.051). All patients received complete resection of the tumor. The mean blood loss in both groups was similar (Table 2).

**Table 2:** Perioperative outcomes

	Jejunum group (*n* = 34)	Colon group (*n* = 37)	*P*-value
**Operative time (minutes)**	397.2 ± 107.1	415.8 ± 123.8	0.5^a^
**Volume of drainage (ml)**	720 (417.5–1355.0)	1055 (672.5–1535.0)	0.14^b^
**Time interval between gastrectomy and esophagectomy (months)**	41.2 ± 9.3	37.4 ± 8.5	0.08 ^a^
**Postoperative drainage time (days)**	5.0 ± 1.6	6.0 ± 2.3	0.04^a^
**Postoperative hospital stay (days)**	12.7 ± 3.3	14.2 ± 4.3	0.12^a^
**Blood loss (ml)**	200 (200.0–462.5)	200 (300–500)	0.65^a^
**Number of lymph nodes dissected**	9.5 (5.00–13.25)	11 (6.0–14.5)	0.46^b^

^a^ Independent Sample T-test

^b^ Mann–Whitney’s U-test

### Postoperative outcomes

There were no significant differences between these two groups in terms of the volume of postoperative drainage, postoperative hospital stay, and total lymph node dissection (Table 2). The mean duration of drainage in the jejunum group was significantly lower than that in the colon group (5.0 ± 1.6 days vs. 6.0 ± 2.3 days, *P* = 0.04). One patient in the jejunum group died on the 6th postoperative day due to anastomotic leakage and secondary lung infection. No patient died in the colon group. Ten patients in the colon group developed anastomotic leakage, which was significantly higher than that in the jejunum group (27% vs. 5.9%, *P* = 0.02). The intrathoracic anastomotic leaks were managed by thorough debridement of the necrotic tissues in the chest, placement of a wide-bore drainage tube, antibiotics and nutritional support. For cervical anastomotic leaks, the neck wound was opened and cleaned, a drainage tube was placed, and antibiotics were administered. There were no significant differences between these two groups in terms of complications, such as postoperative fever and pneumonia (Table 3). However, the incidence of other complications was significantly higher in the colon group than in the jejunum group (32.4% vs. 11.8%, *P* = 0.03). On subgroup analysis of postoperative complications based on the site of anastomosis, no significant differences were noticed (Table 4). For patients with mediastinal anastomosis, or mid- or lower-thoracic tumors, the incidence of anastomotic leakage and halitosis was significantly higher in the colon group (Table 5).

**Table 3:** Postoperative complications

	Jejunum (*n* = 34)	Colon (*n* = 37)	*P*-value
**Fever**	5(14.7%)	7(18.9%)	0.63^a^
**Pneumonia**	3(8.8%)	4(10.8%)	0.78^b^
**Hydrothorax**	3(8.8%)	3(8.1%)	0.91^b^
**Anastomotic leakage**	2(5.9%)	10(27%)	0.02^a^
**Stump fistula**	1(2.9%)	2(5.4%)	0.63^a^
**Halitosis**	6(17.6%)	15(40.5%)	0.03^a^
**Albumin reduction**	17(50%)	19(51.4%)	0.90^a^
**Anemia**	2(5.9%)	3(8.1%)	0.71^a^
**Chest tightness**	0	2(5.4%)	0.49^c^
**Others**	4(11.8%)	12(32.4%)	0.04^a^
**Death**	1(2.9%)	0	> 0.99^c^

^a^ χ^2^ test

^b^ Continuity correction χ^2^ test

^c^ Fisher’s exact test

**Table 4:** Comparison of the perioperative outcomes of the two groups based on the site of anastomosis

	Jejunum (*n* = 34)	Colon (*n* = 37)	*P*-value
**Anastomotic leakage**			0.46 ^a^
Neck	0	6	
Mediastinum	2	4	
**Fever**			0.64 ^a^
Neck	1	2	
Mediastinum	4	5	
**Halitosis**			0.52 ^a^
Neck	3	9	
Mediastinum	3	6	
**Postoperative hospital stay (days)**			
Neck	5.16 ± 1.47	5.10 ± 1.22	0.58 ^b^
Mediastinum	5.0 ± 1.71	6.42 ± 2.60	0.11 ^b^
**Operative time (minutes)**			
Neck	484.2 ± 159.8	394.5 ± 89.1	0.19 ^b^
Mediastinum	378.6 ± 85.2	422.1 ± 134.2	0.06 ^b^
**Blood loss (ml)**			
Neck	433 ± 225	270 ± 298	0.96 ^b^
Mediastinum	333 ± 240	407 ± 246	0.83 ^b^

^a^ χ^2^ test

^b^ Independent Sample T-test

**Table 5:** Comparison of the postoperative complications of the jejunal and colonic conduit in patients with mediastinal anastomosis

	Jejunum (*n* = 24)	Colon (*n* = 19)	*P*-value
**Fever**	3(12.5%)	5(26.3%)	0.25
**Pneumonia**	2(8.3%)	2(10.5%)	0.81
**Hydrothorax**	2(8.3%)	3(15.8%)	0.45
**Anastomotic leakage**	0(5.9%)	7(36.8%)	< 0.01
**Stump fistula**	1(4.2%)	1(5.3%)	0.87
**Halitosis**	3(12.5%)	11(57.9%)	< 0.01
**Albumin reduction**	11(45.8%)	9(47.4%)	0.92
**Anemia**	2(8.3%)	1(5.3%)	0.70
**Chest tightness**	0	2(10.5%)	0.10

χ^2^**-**test was used for the above data

**Fig. 2:**
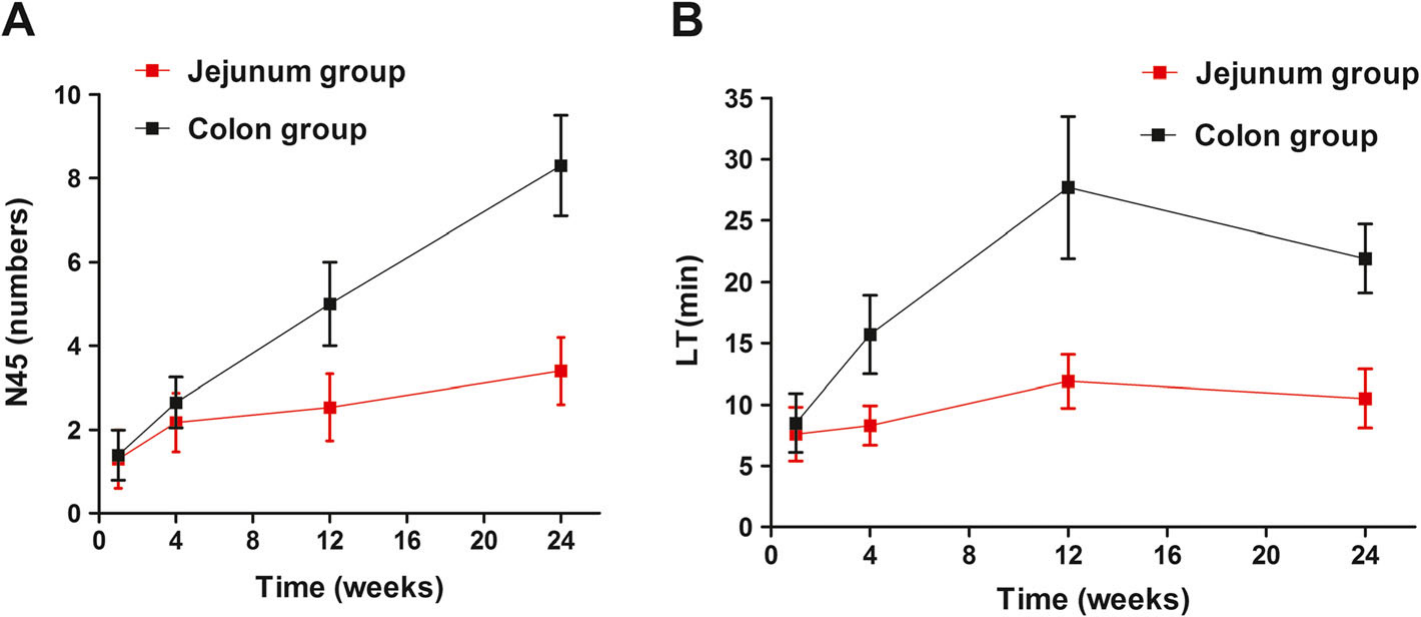
Postoperative acid reflux in patients with jejunal and colonic conduits measured using a pH meter. The jejunal group had significantly lower numbers of pH < 4 simultaneous reflux time > 5 min (N45) (**a**) and the longest reflux time (LT) (**b**) at 24 weeks, when compared to the colon group

Nine patients had no reflux at 24 weeks after surgery, while the remaining 61 patients had varying degrees of reflux, which were treated by medical therapy. At the first week after surgery, there was no difference in N45 and LT between the two groups. After 1 week, N45 and LT rapidly increased in the colon group, but slowly increased in the jejunum group, and at 4 weeks, the differences between the colon group and jejunum group gradually became statistically significant (N45 [2.65 ± 0.6 vs. 2.17 ± 0.7, *P* < 0.05] and LT [15.7 ± 3.2 vs. 8.3 ± 1.6, *P* < 0.05], respectively; Fig. 2). At 24 weeks, the N45 and LT values were significantly higher in the colon group than in the jejunum group (*P* < 0.01).

**Fig. 3:**
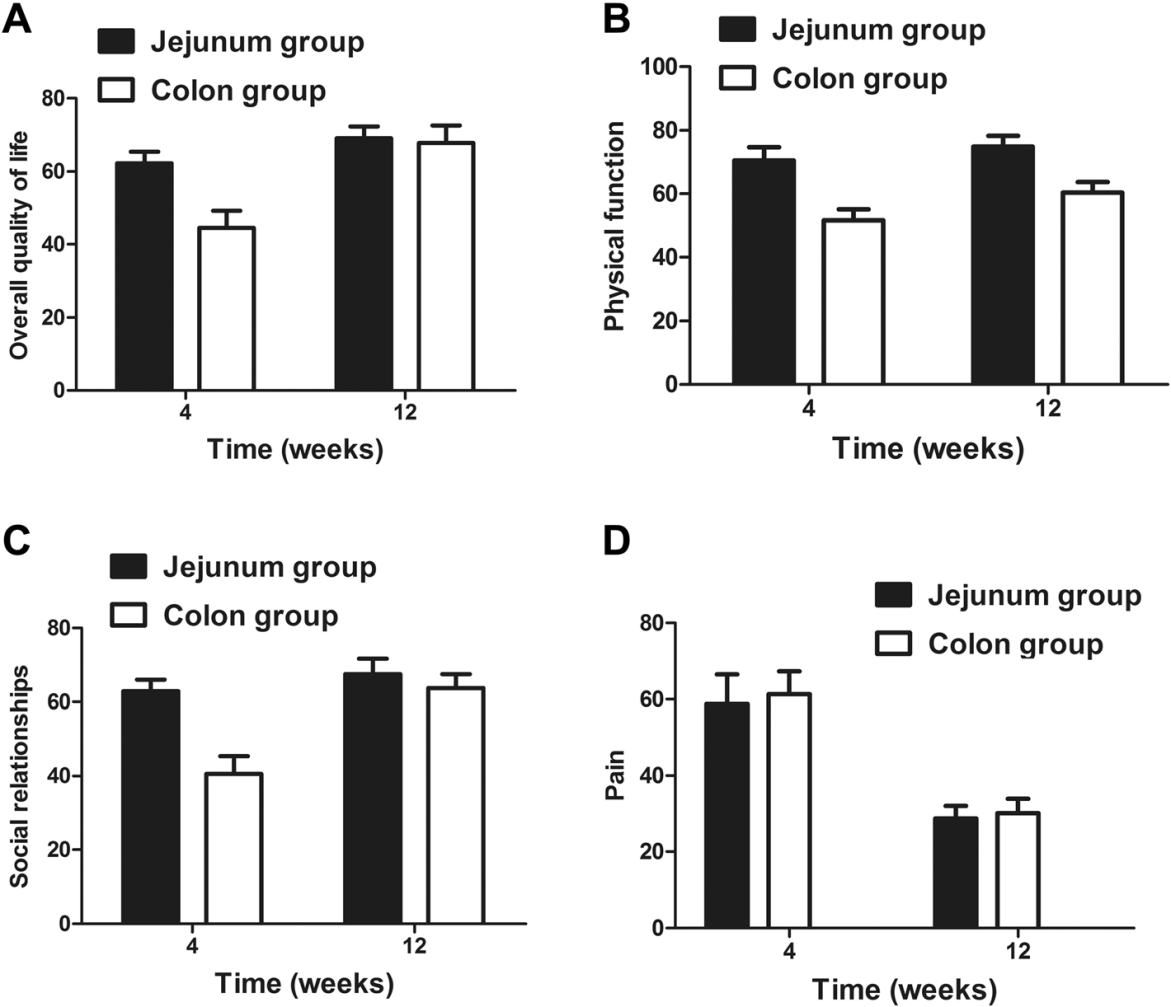
Comparison of postoperative quality of life (QLQ-C30) questionnaire scores in patients who received jejunal and colonic conduits: (**a**) overall quality of life, (**b**) physical functions, (**c**) social relationships, and (**d**) pain scores. The error bars represent the standard deviation

**Fig. 4:**
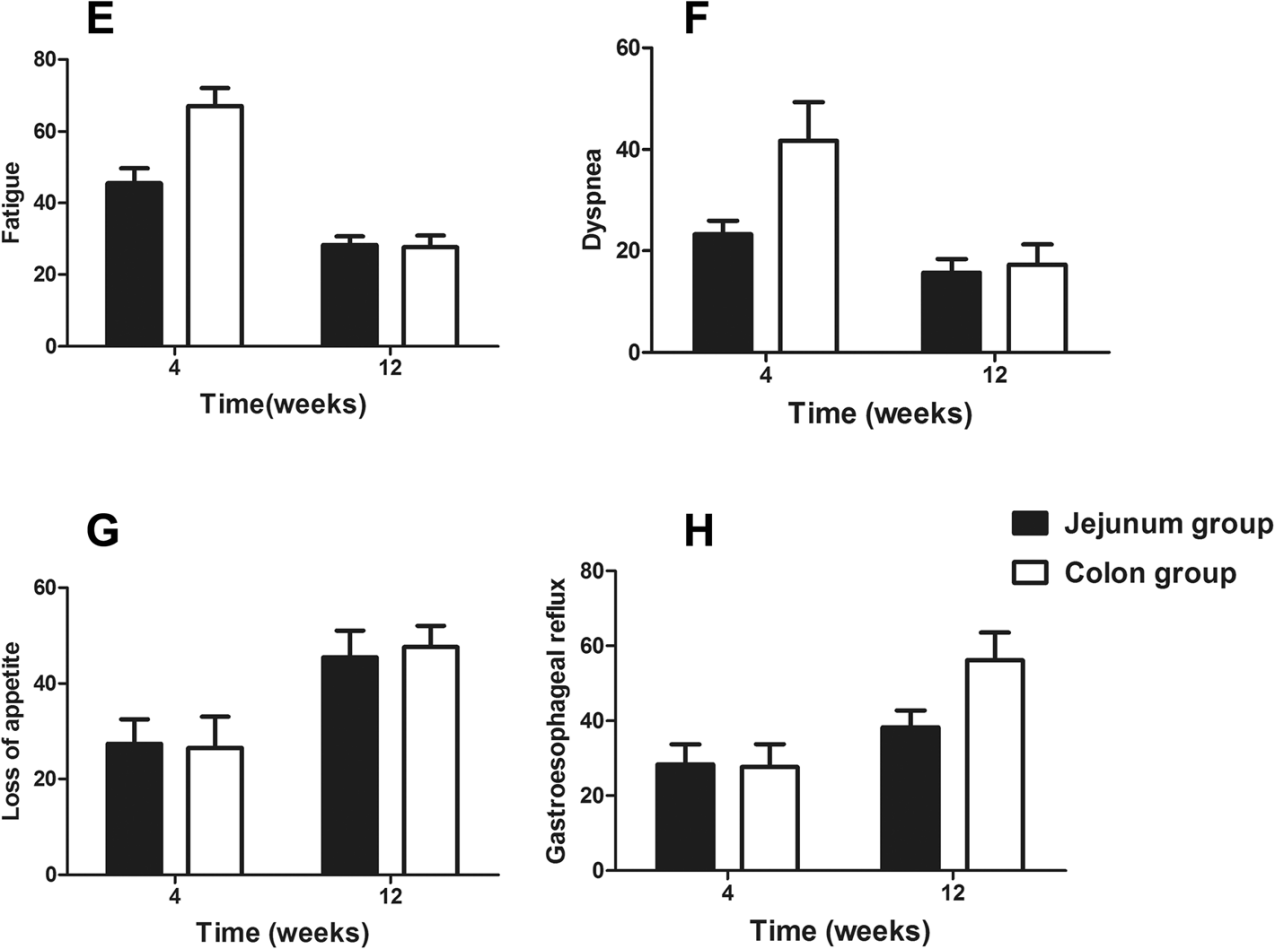
Comparison of postoperative quality of life (QLQ-OES18) supplemental scale scores in patients who received jejunal and colonic conduits: (**a**) fatigue score, (**b**) dyspnea score, (**c**) loss of appetite score, and (**d**) gastroesophageal reflux score

All patients completed the QLQ-C30 and QLQ-OES18 questionnaires at four and 12 weeks after surgery (Supplementary Table S1). It was found that from the 4th week to the 12th week, the functional indicators of both groups gradually recovered, and the symptom index gradually decreased. The overall QOF, physical functions and social relationships were significantly higher in the jejunum group, when compared to the colon group, at 4 weeks after surgery (*P* < 0.05). At 12 weeks after surgery, the social relationships, physical functions and overall QOL of patients in the colon group improved, but these were significantly lower than that in the jejunum group (*P* < 0.05, Fig. 3). For the QLQ-OES18 questionnaire, it was observed that the two groups tended to improve, in terms of dysphagia, dryness of mouth and language difficulties. However, the colon group had a significantly higher incidence of gastroesophageal reflux symptoms, when compared with the jejunum, after the 12th week (38.3 ± 4.5 vs. 56.1 ± 7.4, *P* < 0.05; Fig. 4).

## Discussion

The number of patients with esophageal cancer after gastrectomy is gradually increasing in clinical practice. One of the most important factors may be that the remnant stomach and distal esophagus are constantly exposed to gastric acid and bile reflux, thereby increasing the risk of cancer [21, 22]. The investigators started performing pedicled jejunal esophageal reconstruction in 2005. However, prior to that, the investigators have been performing pedicled colonic conduits for esophageal reconstruction. Studies that compared the outcomes of a pedicled jejunal conduit with a colonic conduit remains limited. In addition, for different patients with different tumor locations, the most suitable conduit remains uncertain. The present study, which had a relatively higher number of patients, attempted to compare the similarities and differences between these two conduits, in terms of perioperative outcomes and postoperative QOL.

In the present retrospective study, pedicled jejunal replacement was found to be superior to a colonic conduit for esophagus reconstruction. There were 12 cases of anastomotic leakage in the present study, which included 10 cases in the colon group, and two cases in the jejunum group. The incidence of anastomotic leak and other complications was lower in the jejunum group. Haubrich [23] proposed that there is higher intestinal bacterial load in colons with strong reproductive ability, and that this may be an important factor that affects the healing of the anastomosis. Hence, the investigators considered that good vascularity and lower bacterial contamination of the jejunum could be responsible for the better outcomes and higher survival rate [24]. However, one of the limitations of a pedicled jejunal conduit was its limited length and mobility, making it unsuitable for reconstruction for patients with upper esophageal tumors. In contrast, adult colonic length is 130–150 cm, with good mobility and strong acid resistance [25]. Hence, for upper thoracic esophageal tumors, a colonic conduit is preferred.

After the esophagectomy, one of the major concerns in the postoperative period is acid reflux. Most postoperative patients have varying degrees of acid reflux [26]. The causes of this phenomenon may be as follows: (1) the absence of a lower esophageal sphincter, (2) peristaltic dysfunction and diminished digestive function of the remnant stomach, and (3) altered esophageal and gastric remnant physiological functions due to vagal denervation during surgery [27]. On the 24-h pH monitoring of the remnant esophagus at 1 week after surgery, there were no significant differences in reflux trends between the two groups. However, at 4 weeks after surgery, N45 and LT were substantially higher in the colon group than in the jejunum group. This was mainly because the jejunum has good peristalsis, which facilitates the effective clearing of the jejunal conduit and reduces the acid reflux into the remnant esophagus. The reflux phenomenon after esophageal reconstruction was found to seriously affect the QOL of patients after surgery. It is noteworthy that the main cause of death of the patient in the jejunum group was malnutrition. Poor physical performance status before surgery led to poor respiratory efforts and respiratory infections. However, based on a single death, it cannot be concluded whether a jejunal conduit is inferior to a colonic conduit.

The QOL assessment through the QLQ-C30 and QLQ-OES18 questionnaires at 4 and 12 weeks revealed that the postoperative QOL of patients in these two groups improved with time. The overall QOL in the jejunum group was higher at 4 weeks, but this became similar to that in the colon group at 12 weeks. This indicates that the recovery of patients with a colonic conduit is slower. Furthermore, it was found that the social relationships of patients in the colon group were lower, when compared to the jejunum group [13]. One of the important responsible factors for this observation was the occurrence of halitosis after surgery, which was significantly higher in the colon group (17.6% vs. 40.5%, *P* = 0.03). In addition, the symptoms of acid reflux were significantly higher at 12 weeks in the colon group. This could be due to the better peristalsis of the jejunum, as opposed to the colon, which facilitates the rapid clearance of acid and food contents, leading to fewer symptoms. However, longer follow-up studies are required to understand the impact of the type of conduit on QOL.

There were some limitations in the present study. First, the sample size was relatively small, and the study was retrospective in nature. Second, this was a single-center study. Furthermore, the data at our center could not be combined with the data from other centers due to significant differences in perioperative patient care and surgical methods. Third, there was some heterogeneity in the two groups with regards to age, tumor location and other factors. Fourth, due to the long study period and use of different adjuvant therapies in the two groups, the long-term survival data could not be compared. Due to length constraints in upper esophageal tumors that require a high cervical anastomosis, in which a jejunal conduit could not be used, a fair comparison between the types of conduits was not feasible. Future prospective randomized studies for mid and lower esophageal tumors are needed to compare the outcomes of jejunal and colonic conduits without selection bias. Furthermore, the postoperative QOL assessment in the present study had a short follow-up interval, and received different postoperative radiotherapy or chemotherapy treatments. Therefore, the investigators specifically followed up the data before the postoperative adjuvant therapy to reduce the impact of adjuvant therapy on the study data, and reflect the true differences between the two groups.

## Conclusions

In summary, for patients with previous gastrectomy having esophageal cancer, the use of a pedicled jejunal conduit after esophagectomy is associated with a lower incidence of anastomotic leaks and other complications, lower acid reflux, and higher short-term QOL. This may be considered as the first choice of conduit, especially for mid and lower esophageal tumors, in which a very long conduit for cervical anastomosis is not required. When the tumor is in the upper esophagus and the jejunal conduit cannot reach high up for the reconstruction, the colon can be used to complete the reconstruction of the digestive tract.

### Supplementary information

**Additional file 1: Supplementary Figure 1** This figure is intended for the neck anastomosis operation. The pedicle of the pedicle will be released as much as possible to meet the length of the neck.

**Additional file 2: Supplementary Figure 2** Intraoperative photographs: (a) The selected jejunal segment for esophageal reconstruction. The *arrow* indicates the vascular arch retained during surgery; (b) The esophagojejunostomy (arrow) is being performed.

**Additional file 3.**

**Additional file 4.**

## Data Availability

The datasets used and/or analyzed during the present study are available from the corresponding author on reasonable request.

## The appendix

***(1) Jejunum group:*** First, the thoracic esophagus was mobilized along its longitudinal axis. The first jejunal vessels were identified and preserved to maintain the blood supply to the proximal jejunum. Then, the 2nd, 3rd and 4th jejunal vessels found on the main vascular arch were occluded using a vascular clamp for more than 10 min, in order to observe the blood supply. If the small jejunal arteries in the clamping area continue to pulsate, or the jejunum does not become discolored, this suggests that the blood supply is good. Then, the occluded jejunal vessels were clipped and divided to facilitate the lifting up of the jejunum (Supplementary Figure 1). In most cases, the 4th jejunal branch of the superior mesenteric artery functions as the distal vascular pedicle to the mobilized jejunal conduit. The jejunum was divided at approximately 20 cm from the ligament of Treitz. Generally, the length of the jejunal tube is sufficient to reach the mid to upper chest. Subsequently, the tumor bearing segment of the esophagus was resected. The proximal esophago-jejunal anastomosis was completed in an end-to-end fashion using the hand-sewn technique with two layers of sutures, or in an end-to-side fashion using a stapling device. If the tumor was located in the upper esophagus, a cervical incision was made along the left sternocleidomastoid, and the cervical esophagus was mobilized. The jejunal conduit was mobilized to reach up to the neck via the esophageal bed, and the esophago-jejunal anastomosis was completed in the neck. The jejunal conduit was divided in the abdomen at an appropriate length, and the distal end of the jejunum was anastomosed to the anterior wall of the gastric remnant anastomosis in an end-to-side fashion. Lastly, an end-to-side jejuno-jejunal anastomosis was carried out to restore digestive tract continuity (Supplementary Figure 2). The jejunal conduit was partly angulated due to short mesentery, as presented in other Roux-en-Y procedures.

***(2) Colon group*****:** When using the colon as an esophageal substitute, the most critical step is to choose the appropriate vascular pedicle and mobilize the corresponding colonic segment. Generally, the length of the colon is measured based on the length of the mesenteric vascular arch, and the length of the conduit should preferably be approximately 3 cm longer than the desired length. For mobilization, first, the hepatocolic ligament and gastrocolic ligament are divided, and the hepatic flexure, transverse colon and splenic flexure are mobilized. Second, when the left colonic graft is used, the ascending branch of the left colic artery is used as a vascular pedicle, and the left branch of the middle colon artery is ligated. However, if the right colonic graft is used, the middle colic artery is used as the nutrient vessel of the graft, and the ileocolic and right colic arteries are ligated. For the construction of the colonic grafts, the vascular clamp test was performed for 15 min before dividing the colonic vessels. The colonic vessels were ligated only when the blood vessels at the edge of the colonic conduit had palpable pulsations, and the color of the conduit remained unchanged after clamping for 15 min. Finally, the esophago-colonic and colo-gastric remnant and colo-colic anastomosis were performed similar to that described for the pedicled jejunal graft.

## Abbreviations

LT: Longest reflux time
QOL: Quality of life
ANOVA: analysis of variance
