# Supplementary figures and images for: Comparison of outcomes of pedicled jejunal and colonic conduit for esophageal reconstruction
Source: BMC Surg. 2020 Jul 16;20:156. doi: 10.1186/s12893-020-00810-y (PMC7364600; doi:10.1186/s12893-020-00810-y)

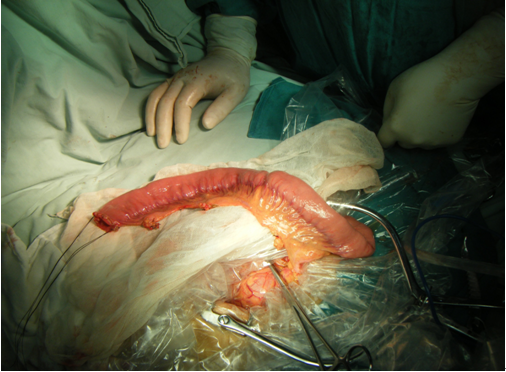

Supplement: Supplementary file 1 — Additional file 1: Supplementary Figure 1 This figure is intended for the neck anastomosis operation. The pedicle of the pedicle will be released as much as possible to meet the length of the neck. [file 12893_2020_810_MOESM1_ESM.tif]

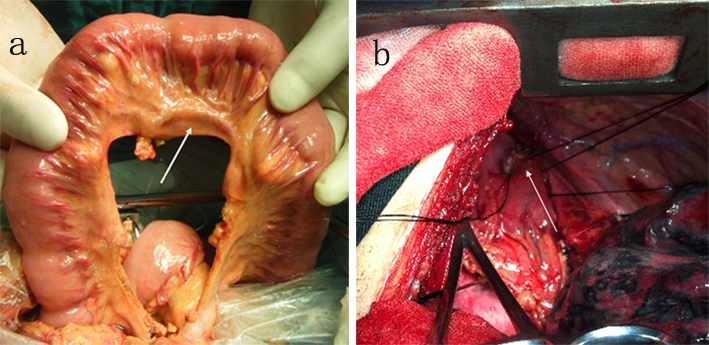

Supplement: Supplementary file 2 — Additional file 2: Supplementary Figure 2 Intraoperative photographs: (a) The selected jejunal segment for esophageal reconstruction. The arrow indicates the vascular arch retained during surgery; (b) The esophagojejunostomy (arrow) is being performed. [file 12893_2020_810_MOESM2_ESM.tif]
