# Supplementary material for: Comparison of outcomes of pedicled jejunal and colonic conduit for esophageal reconstruction
Source: BMC Surg. 2020 Jul 16;20:156. doi: 10.1186/s12893-020-00810-y (PMC7364600; doi:10.1186/s12893-020-00810-y)
Supplement: Supplementary file 3 — Additional file 3. [file 12893_2020_810_MOESM3_ESM.doc]

**The EORTC questionnaire QLQ-C30 (Chinese＆English)**

我们想了解有关您和您的健康的一些情况，请您亲自回答下面所有问题，这里的答案并无“对”与“不对”，只要求在最能反映您情况的那个数字上画圈。您所提供的资料我们将会严格保密,谢谢配合。

***以下问题的后面出现的1、2、3、4分别代表没有、有一点、有一些、非常多***

1.您从事一些费力的活动有困难吗，比如说提很重的购物袋或手提箱？ 1 2 3 4

2.长距离行走对您来说有困难吗？ 1 2 3 4

3.户外短距离行走对您来说有困难吗？ 1 2 3 4

4.您白天需要呆在床上或椅子上吗？ 1 2 3 4

5.您在吃饭、穿衣、洗澡或上厕所时需要他人帮忙吗？ 1 2 3 4

在过去的一星期内：

6．您在工作和日常活动中是否受到限制？ 1 2 3 4

7．您在从事您的爱好或休闲活动时是否受到限制? 1 2 3 4

8．您有气促吗？ 1 2 3 4

9．您有疼痛吗？ 1 2 3 4

10．您需要休息吗？ 1 2 3 4

11．您睡眠有困难吗？ 1 2 3 4

12．您觉得虚弱吗？ 1 2 3 4

13．您食欲不振（没有胃口）吗？ 1 2 3 4

14．您觉得恶心吗？ 1 2 3 4

15．您有呕吐吗？ 1 2 3 4

16．您有便秘吗？ 1 2 3 4

在过去的一星期内：

17．您有腹泻吗？ 1 2 3 4

18．您觉得累吗？ 1 2 3 4

19．疼痛影响您的日常活动吗？ 1 2 3 4

20．您集中精力做事有困难吗，如读报纸或看电视？ 1 2 3 4

21．您觉得紧张吗？ 1 2 3 4

22．您觉得忧虑吗？ 1 2 3 4

23．您觉得脾气急躁吗？ 1 2 3 4

24．您觉得压抑（情绪低落）吗？ 1 2 3 4

25．您感到记忆困难吗？ 1 2 3 4

26．您的身体状况或治疗影响您的家庭生活吗？ 1 2 3 4

27．您的身体状况或治疗影响您的社交活动吗？ 1 2 3 4

28．您的身体状况或治疗使您陷入经济困难吗？ 1 2 3 4

对下列问题，数字1至7代表从“很差”到“很好”的等级。请在1-7之间选出一个最适合您的数字并画圈。

29．您如何评价在过去一星期内您总的健康情况？

1 2 3 4 5 6 7

30．您如何评价在过去一星期内您总的生命质量？

1 2 3 4 5 6 7

We would like to know some things about you and your health. Please answer all the questions below. There are no “right” or “wrong” answers. Encircle the answer that best reflects your situation. The information you provide will be kept strictly confidential. Thank you for your cooperation.

***The numbers 1, 2, 3 and 4 that appear after each question represent the following: no, a little, some, and many.***

1. Is it difficult for you to engage in some laborious activities, such as carrying a heavy shopping bag or suitcase?

1 2 3 4

2. Is it difficult for you to travel long distances?

1 2 3 4

3. Is it difficult for you to walk outdoors for short distances?

1 2 3 4

4. Do you need to stay in bed or a chair during the day?

1 2 3 4

5. Do you need help from others when eating, dressing, bathing, or going to the bathroom?

1 2 3 4

In the past week:

6. Are you restricted in your work and daily activities?

1 2 3 4

7. Are you restricted when engaging in your hobbies or leisure activities? 1 2 3 4

8. Do you have shortness of breath? 1 2 3 4

9. Do you have pain? 1 2 3 4

10. Do you need a break? 1 2 3 4

11. Are you having trouble sleeping? 1 2 3 4

12. Do you feel weak? 1 2 3 4

13. Are you losing your appetite (no appetite)? 1 2 3 4

14. Do you feel sick? 1 2 3 4

15. Do you feel like vomiting? 1 2 3 4

16. Do you have constipation? 1 2 3 4

In the past week:

17. Do you have diarrhea? 1 2 3 4

18. Do you feel tired? 1 2 3 4

19. Does pain affect your daily activities? 1 2 3 4

20. Do you have trouble concentrating on doing things, such as reading a newspaper or watching TV? 1 2 3 4

21. Do you feel nervous? 1 2 3 4

22. Do you feel worried? 1 2 3 4

23. Do you feel tempered? 1 2 3 4

24. Do you feel depressed (low mood)? 1 2 3 4

25. Do you find it difficult to remember things? 1 2 3 4

26. Does your physical condition or treatment affect your family life?

1 2 3 4

27. Does your physical condition or treatment affect your social activities?

1 2 3 4

28. Does your physical condition or treatment give you financial difficulties?

1 2 3 4

***For the following questions, numbers 1 to 7 represent the levels from "very poor" to "very good." Please choose and encircle a number between 1 to 7 that best suits you.***

29. How would you rate your overall health over the past week?

1 2 3 4 5 6 7

30. How would you rate your total quality of life in the past week?

1 2 3 4 5 6 7

**The EORTC questionnaire QLQ-OES18 (Chinese＆English)**

请您亲自回答下面所有问题，只要求在最能反映您情况的那个选项上打（**√**）。您所提供的资料我们将会严格保密，谢谢配合。

1.您能够吃固体食物（如干饭和大馍）吗？

完全不能 经常不能 有时不能 基本可以

2.您能够吃稀饭或面汤吗？

完全不能 经常不能 有时不能 基本可以

3.您能够喝水吗？

完全不能 经常不能 有时不能 基本可以

4.您吞口水是否有困难？

完全没有 有点困难 比较困难 很困难

5.您吞咽是有哽噎感吗？

完全没有 有时有 经常有 总是有

6.您是否觉得吃东西是一种享受？

大多数是 有时不是 经常不是 完全不是

7.您是否觉得进食后马上就有饱胀感？

完全没有 有时有 经常有 总是有

8.您是否有进食难受的感觉？

完全没有 有时有 经常有 总是有

9.您在其他人面前进食有感到难为情吗？

完全没有 有时有 经常有 总是有

10.您有嘴巴发干的感觉吗？

完全没有 有一点 比较明显 很明显

11.您有吃饭不香的问题吗？

完全没有 有时有 经常有 总是有

12.您有因为咳嗽感到烦恼吗？

完全没有 有点烦恼 比较烦恼 很烦恼

13.您说话有困难吗？

完全没有 有点困难 比较困难 很困难

14.您有胃酸过多、消化不良或胸口烧灼感吗？

完全没有 有时有 经常有 总是有

15.您曾有胃酸或胆汁逆流到口中的不舒服感吗？

完全没有 有时有 经常有 总是有

16.您有进食时感到有疼痛吗？

完全没有 有点痛 比较痛 很痛

17.您感到过有胸部疼痛吗？

完全没有 有时有 经常有 总是有

18.您感到过有胃痛吗？

完全没有 有时有 经常有 总是有

Please answer all the questions below, and choose the option that best reflects your situation (**√**). The information you provide will be kept strictly confidential. Thank you for your cooperation.

1. Can you eat solid foods (such as dry rice and big rice)?

Not at all often not sometimes not basically

2. Can you eat porridge or noodle soup?

Not at all often not sometimes not basically

3. Can you drink water?

Not at all often not sometimes not basically

4. Do you have any difficulties in swallowing water?

Not at all a little difficult more difficult very difficult

5. Do you have a sensation of swallowing when swallowing?

Not at all sometimes often always

6. Do you feel that eating is a pleasure?

Most are sometimes not often not not at all

7. Do you feel a sense of fullness immediately after eating?

Not at all sometimes often always

8. Do you feel uncomfortable eating?

Not at all sometimes often always

9. Do you feel embarrassed when you eat with other people?

Not at all sometimes often always

10. Do you have a dry mouth feeling?

Not at all a little obvious more obvious obviously

11. Do you also have a problem with eating?

Not at all sometimes often always

12. Do you have trouble with coughing?

Not at all a little trouble more trouble very troubled

13. Is it difficult to speak?

Not at all a little difficult more difficult very difficult

14. Do you have too much stomach acid, indigestion, or a burning sensation in your chest?

Not at all sometimes often always

15. Have you ever had an uncomfortable feeling of stomach acid or bile flowing back into your mouth?

Not at all sometimes often always

16. Do you feel pain when eating?

No pain at all a little pain more pain a lot of pain

17. Have you ever felt chest pain?

Not at all sometimes often always

18. Have you ever had a stomachache?

Not at all sometimes often always
