# Supplementary material for: Comparison of outcomes of pedicled jejunal and colonic conduit for esophageal reconstruction
Source: BMC Surg. 2020 Jul 16;20:156. doi: 10.1186/s12893-020-00810-y (PMC7364600; doi:10.1186/s12893-020-00810-y)
Supplement: Supplementary file 4 — Additional file 4. [file 12893_2020_810_MOESM4_ESM.doc]

**Supplementary Table S1. Life quality scores of the different esophageal reconstructions at a given time interval**

|  |  | 4 weeks after the operation | | |  | 12 weeks after the operation | | |
| --- | --- | --- | --- | --- | --- | --- | --- | --- |
|  |  | Jejunum | Colon | *p* |  | Jejunum | Colon | *P* |
| **QLQ-C30** | Overall quality of life | 62.2±3.2 | 44.5±4.7 | <0.05 |  | 69.1±3.2 | 67.8±4.8 | 0.23 |
|  | Physical functions | 70.5±4.2 | 51.6±3.5 | <0.05 |  | 74.9±3.4 | 60.4±3.3 | <0.05 |
|  | Role functions | 59.1±3.8 | 57.6±4.4 | 0.16 |  | 68.3±2.8 | 68.1±4.4 | 0.83 |
|  | Emotional functions | 74.2±3.7 | 72.8±4.5 | 0.19 |  | 82.4±2.6 | 82.9±4.2 | 0.58 |
|  | Cognitive functions | 65.4±2.9 | 64.7±3.6 | 0.41 |  | 75.2±3.5 | 75.8±3.2 | 0.48 |
|  | Social relationships | 62.9±3.1 | 40.6±4.8 | <0.05 |  | 67.5±4.2 | 63.8±3.7 | <0.05 |
|  | Fatigue | 45.6±4.1 | 67.2±4.8 | <0.05 |  | 28.2±2.5 | 27.6±3.3 | 0.43 |
|  | Nausea and vomiting | 11.8±3.4 | 12.4±4.6 | 0.57 |  | 5.0±1.2 | 5.2±1.6 | 0.57 |
|  | Pain | 58.8±7.7 | 61.3±6.0 | 0.16 |  | 28.7±3.3 | 30.1±3.8 | 0.13 |
|  | Dyspnea | 23.2±2.7 | 41.8±7.6 | <0.05 |  | 15.7±2.7 | 17.3±4.0 | 0.07 |
|  | Agrypnia | 29.1±6.2 | 30.4±5.3 | 0.38 |  | 18.5±4.1 | 19.3±3.7 | 0.43 |
|  | Loss of appetite | 27.4±5.1 | 26.5±6.6 | 0.54 |  | 45.4±5.6 | 47.6±4.4 | 0.09 |
|  | Constipation | 27.4±5.1 | 26.5±6.6 | 0.65 |  | 17.4±5.1 | 16.7±6.6 | 0.62 |
|  |  | | | | | | | |
| **QLQ-OES18** | Diarrhea | 25.9±5.6 | 26.8±6.2 | 0.56 |  | 17.1±4.4 | 15.2±5.3 | 0.13 |
|  | Economic difficulty | 60.8±9.2 | 62.1±8.4 | 0.57 |  | 54.9±4.8 | 55.7±6.2 | 0.58 |
|  | Dysphagia | 23.8±3.8 | 24.6±4.2 | 0.44 |  | 18.0±3.7 | 17.2±4.9 | 0.47 |
|  | Eating habit | 24.1±4.8 | 26.8±7.9 | 0.11 |  | 22.4±4.4 | 24.1±5.0 | 0.16 |
|  | Acid reflux | 28.4±5.3 | 27.6±6.2 | 0.59 |  | 38.3±4.5 | 56.1±7.4 | <0.05 |
|  | Dry mouth | 18.9±2.4 | 19.7±3.8 | 0.33 |  | 15.6±2.7 | 14.8±2.0 | 0.19 |
|  | Language difficulty | 12.9±1.8 | 12.2±3.1 | 0.28 |  | 6.6±1.9 | 5.7±1.2 | 0.07 |

Note: Each score is presented as mean ± standard deviation (SD).
